# 931. Epidemiology and Clinical Outcomes of *SARS-CoV-2* Infection Among Pediatric Solid Organ Transplant Recipients: A Single-Center Case Experience

**DOI:** 10.1093/ofid/ofab466.1126

**Published:** 2021-12-04

**Authors:** Jesus Sotelo, Esra Akkoyun, Giselle S Chery, Amal Aqul, Dev M Desai, Paul K Sue, Alyssa Power

**Affiliations:** 1 University of Texas Southwestern Medical Center, Dallas, Texas; 2 UT Southwestern Medical Center, Dallas, Texas; 3 UTSW, Dallas, Texas

## Abstract

**Background:**

The impact of Severe Acute Respiratory Syndrome Coronavirus-2 (SARS-CoV-2) infection among pediatric solid organ transplant (SOT) recipients remains unclear. We sought to characterize the clinical epidemiology and outcomes following SARS-CoV-2 infection among pediatric SOT recipients in Dallas, TX.

**Methods:**

Retrospective review of all SOT recipients with laboratory confirmed COVID-19 infection from March 1, 2020 –March 31, 2021. Demographic, clinical, and outcome data were stratified by transplant type and disease severity. Fischer’s exact test and Kruskall-Wallis test were used to evaluate risk factors for more severe disease among hospitalized children.

**Results:**

Twenty-six SOT recipients with a median age of 14 years were included in the study. Fifteen (58%) were female, eighteen (69%) were Hispanic and thirteen (50%) were overweight/obese. Median time post-transplant was 3.6 years (1311 days, interquartile range (IQR) 394-2881). Fourteen patients were liver recipients, seven kidney, three heart, and two multiorgan. The majority of patients (65%) had a known community exposure and presented with fever (50%), cough (38%) and GI symptoms (19%). Half of all cases were hospitalized (n=13), with 2 requiring intensive care unit (ICU) admission, but no patients required positive pressure ventilation. Median hospital stay was 3 days. Five of the thirteen hospitalized patients were categorized as having moderate disease. No patients developed severe disease and there were no deaths. Older children, as well as children with multiple co-morbidities were noted on univariate analysis to be at higher risk for moderate, as compared to mild, disease.

**Conclusion:**

SARS-CoV-2 infection among pediatric SOT recipients are at increased risk for hospital admission but demonstrate an overall mild /moderate disease course. Larger studies are required to elucidate the risk of morbidity between pediatric SOT recipients and immunocompetent children with SARS-CoV-2.

**Disclosures:**

**Amal Aqul, MD**, **Albireo pharma Inc.** (Consultant)

